# Informing ‘good’ global health research partnerships: A scoping review of guiding principles

**DOI:** 10.1080/16549716.2021.1892308

**Published:** 2021-03-11

**Authors:** Erynn M. Monette, David McHugh, Maxwell J. Smith, Eugenia Canas, Nicole Jabo, Phaedra Henley, Elysée Nouvet

**Affiliations:** a University of Western Ontario, London, Canada; b School of Kinesiology, Faculty of Health Sciences, University of Western Ontario, London, Canada; c School of Health Studies, Faculty of Health Sciences, University of Western Ontario, London, Canada; d Faculty of Information and Media Studies, University of Western Ontario, London, Canada; e Center for One Health, University of Global Health Equity, Butaro, Rwanda

**Keywords:** Equity, fairness, transnational, international, values, guidelines

## Abstract

**Background**: Several sets of principles have been proposed to guide global health research partnerships and mitigate inequities inadvertently caused by them. The existence of multiple sets of principles poses a challenge for those seeking to critically engage with and develop their practice. Which of these is best to use, and why? To what extent, if any, is there agreement across proposed principles?

**Objective**: The objectives of this review were to: (1) identify and consolidate existing documents and principles to guide global health research partnerships; (2) identify areas of overlapping consensus, if any, regarding which principles are fundamental in these partnerships; (3) identify any lack of consensus in the literature on core principles to support these partnerships.

**Methods**: A scoping review was conducted to gather documents outlining ‘principles’ of good global health research partnerships. A broad search of academic databases to gather peerreviewed literature was conducted, complemented by a hand-search of key global health funding institutions for grey literature guidelines.

**Results**: Our search yielded nine sets of principles designed to guide and support global health research partnerships. No single principle recurred across all documents reviewed. Most frequently cited were concerns with mutual benefits between partners (n = 6) and equity (n = 4). Despite a lack of consistency in the inclusion and definition of principles, all sources highlighted principles that identified attention to fairness, equity, or justice as an integral part of good global health research partnerships.

**Conclusions**: Lack of consensus regarding how principles are defined suggests a need for further discussion on what global health researchers mean by ‘core’ principles. Research partnerships should seek to interpret the practical meanings and requirements of these principles through international consultation. Finally, a need exists for tools to assist with implementation of these principles to ensure their application in research practice.

## Background

Research partnerships between institutions in the Global South and the Global North are often challenged by power dynamics and resource differences [1–3]. Different expectations [4], cultural and institutional norms [5,6], logistical and technical communication issues [6,7], and inequitable access to and sharing of resources [1–3,8,9] exacerbate these challenges and pose significant threats to the success of these partnerships [10]. In an effort to mitigate inequities often inadvertently caused by partnerships of this nature [11], professionals working in the global health space have started to engage in discussions of what ethical global health research partnerships look like in practice and theory [4,8,9]. Researchers and research institutions are increasingly confronted with questions of what values, outcomes, or practices must exist within their own transnational partnerships to ensure they are successful in reducing global health inequities within their projects and teams, but also more broadly.

In recent years, several organizations and author groups have developed documents proposing theoretical principles aimed at reducing partnership inequities [10,12,13]. In each of these guiding documents, the authors include a series of commitments and considerations they regard as integral to supporting equity and mitigating power and resource disparities in global health research partnerships. While each of these contributions is valuable, the existence of so many guiding documents presents a challenge for individuals, teams, or organizations seeking to critically engage with and develop their own global health research partnerships. Which set of principles, or guiding documents, if any, should one use? Is there overlapping consensus about the sorts of values and commitments one ought to have when engaging in global health research partnerships? Why use these guiding documents at all?

This article provides a scoping review of key principles available (as of February 2020) to support global health research partnerships and their evaluation. In doing so, our goals are threefold: (1) identify and consolidate existing documents and principles developed to guide global health research partnerships; (2) identify areas of overlapping consensus, if any, regarding which principles are fundamental in global health research partnerships; and (3) identify any lack of consensus in the literature on core principles to support global health research partnerships. Ultimately, we aim to facilitate awareness, use, and potential refinement of these guiding documents amongst global health research practitioners.

### Defining ‘global health research partnerships’

A ‘global health research partnership’ can be defined as any global health research project that involves collaboration between investigators or institutions in two or more countries. The partnerships we are particularly interested in here are those that exist between nations in the ‘Global North’ (sometimes referred to as ‘higher-income countries’ (HICs)) and ‘Global South’ (sometimes referred to as ‘low- and middle-income countries’ (LMICs)) [14]. While these distinctions arose from the observed trend that the majority of HICs are located in the Northern Hemisphere and the majority of LMICs in the Southern Hemisphere, they do not always reference geographical north and south [14]. Rather, they refer primarily to the presence of economic power dynamics between nations [2]. Global North countries can be described as those holding significant financial and logistical resources. Consequently, countries and organizations in the Global North often act as major sponsors of research and supply financial, technical, educational, and in many cases, personnel resources [1,2]. Due to the fact that nations in the Global North frequently hold the majority of resources necessary to conduct research in the global health space, they often also hold power over how, where, and when research is conducted. This reality persists even in instances where research is conducted and co-led by researchers in the Global South [2]. Although our focus was on Global South and Global North partnerships, we also reviewed Global North/Global North and Global South/Global South partnerships in global health research.

## Methods

Guiding documents outlining principles for global health research were gathered using a modified version of Arksey and O’Malley’s scoping review methodology [15]. As summarized in Table 1, our search strategy gathered sources featuring principles of global health research in two phases: (1) a search of academic literature, and (2) a hand-search of key global health funding agencies and organization websites for grey resources. The keywords ‘global,’ ‘international,’ ‘health,’ ‘partnerships,’ ‘research,’ ‘principles,’ ‘guidelines,’ ‘framework,’ and ‘model’ were used to limit results to answer our specific question. We defined ‘principles’ as any word, phrase, or recommendation that is listed as a guiding statement or value that is proposed as being integral to global health research partnerships. In this review, we included sources that met our definition of global health principles even if sources did not explicitly refer to them as such (for example, Larkan, Uduma, Lawal, and van Bavel [7] refer to these as ‘core concepts’; Raza [16] refers to them as ‘essential ingredients’).

**Table 1.** Search strategy answering the question: ‘What sets of principles have been developed to inform *equitable global health research partnerships*?’

	Search concepts combined using Boolean operator ‘AND’				
	*Concept #1*	*Concept #2*	*Concept #3*	*Concept #4*	*Concept #5*
**Keywords combined using Boolean operator ‘OR’**	global	partnerships	research	health	guidelines
	international	collaboration			principles
					model
					framework

**Figure 1.**
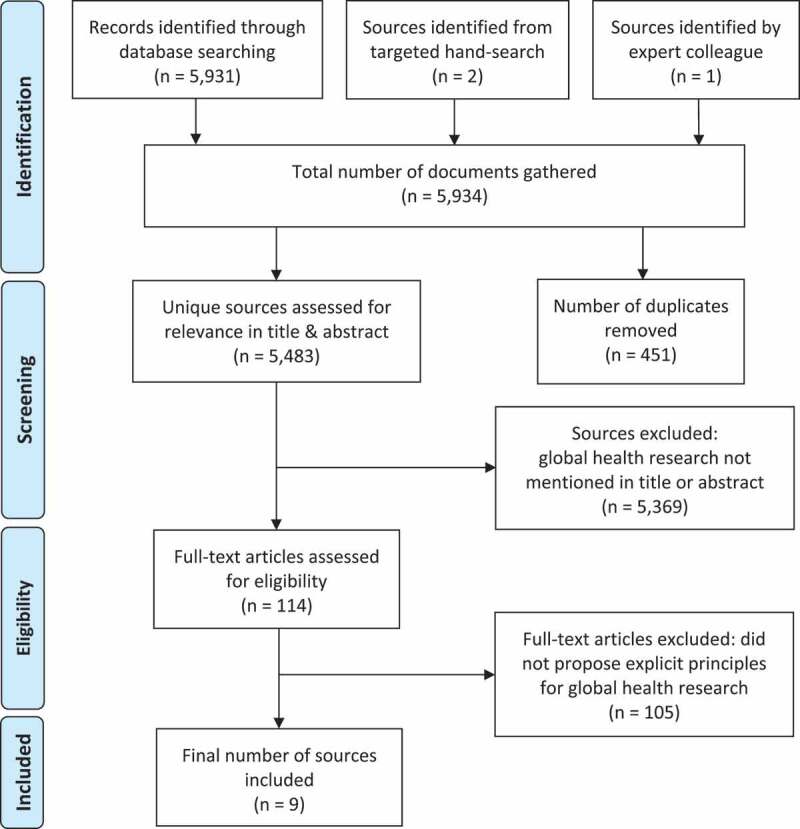
PRISMA [18] diagram of the review process producing the nine sources included in this review

In the first phase of this review, relevant academic literature was identified using the databases PubMed, Web of Science, and Scopus. The search of these databases returned 5,931 potential sources collectively. Using the inclusion/exclusion criteria outlined in Table 2, the titles and abstracts of these sources were reviewed to ascertain relevance. After removal of duplicates, this process reduced sources for potential inclusion to 114 (see Figure 1). Two reviewers independently performed a full-text review of these 114 sources. Both reviewers identified the same seven sources as meeting inclusion criteria through this process.

**Table 2.** Summary of inclusion and exclusion criteria with rationale

Inclusion Criteria	Exclusion Criteria	Rationale
Principles must be specifically presented as applicable in a ‘global’ or ‘international’ health context.	Principles are not specified to guide ‘global’ or ‘international’ health partnerships.	This review is meant to inform principles to guide transnational partnerships specifically. Although some guidelines for community or local projects may be deduced as applicable to global health projects, those not specifically applied in an international context were considered out of the scope of this paper.
Principles must be intended to guide research practice.	Principles that are indicated to guide non-research global health programs were not included.	The scope of this paper is to review principles that are intended to have relevance to global health research partnerships and practice.

The drop in included literature from the initial search was drastic, but not surprising given the specificity of our review objective. Many of the sources initially retrieved outlined principles for global health partnerships, but only those focused specifically on research partnerships were retained. Since sources discussing global health partnerships outside of research settings – for example for business, non-governmental development activities, or education – fell outside of the scope of this paper, they were ultimately excluded.

In the second phase of this review, we aimed to identify any additional sets of principles meeting inclusion criteria not captured in the database, but publicly available as grey literature sources. This second phase consisted of hand-searching a list of websites of major global health organizations and funding agencies. This list was developed in consultation with a Canadian-Rwandan team of global health researchers familiar with the global health funding landscape. One reviewer hand-searched the websites of the 18 major funders of global health research identified through this consultative process (Appendix A). This targeted hand-search yielded two sources meeting inclusion criteria (see Table 1). One out of these two had been captured through the original scoping database search, so that ultimately only one new source was retained for review from this second phase. Finally, outside these intentional searches, one additional source [17] was recommended by an expert colleague. At the time of recommendation, this source was not yet archived by any organization or database but had recently been referenced on an organizational blog. Given its fit with inclusion criteria, it was included in the final pool of sources for review.

## Results

### Overview

Our search identified nine documents outlining principles that informed global health research partnerships. Five of these were guidelines developed by leading organizations and funders of global health research [11,17,19–21] (three sources were identified in the database search [11,20,21], one source was identified by the targeted hand-search [19], and one source was recommended by an expert colleague [17]). The process of development and the level of detail included in the guiding documents differed across each set of principles. The Netherlands Development Assistance Research Council (RAWOO) [21], for example, included only three overarching principles. In contrast, the Swiss Commission for Research Partnerships with Developing Countries’ (KFPE) [20] ‘A Guide for Transboundary Research Partnerships’ outlined 11 principles, which were accompanied by seven questions to assist partners in critical reflection. A list of all guiding principles proposed in each of these sources and their definitions is available in Appendix B.

While all documents reviewed share a common overarching purpose to support ‘good practice’ and reduce inequities in global health research partnerships, different sets of principles had different foci and justifications. Four of nine guiding documents defined equity and fairness as the primary goal of their proposed principles [7,11,20,21]. Two sets of principles emerged with the more specific interest of improving relationships between global health research partners [16,22]. Raza [16] stated that the purpose of their principles was to summarize ‘basic factors that are required for forging collaboration and responsible attitudes to sustain the relationship’ [16, p.177]. Costello and Zumla [22] proposed that their principles must be present in any ‘truly cooperative’ [22, p.828] partnership. Further, Costello and Zumla [22] suggested that their principles could assist partnerships in moving away from asymmetric power dynamics to avoid negative consequences that outweigh beneficial research outcomes. Two sets of principles focused on improving general research practice and strengthening capacity in specific sectors [10,23]. Steenhoff, Crouse, Lukolyo, Larson, Howard, Mazhani et al. [24] developed principles tailored specifically for global child health research practice, while the Rethinking Research Collaborative (RRC) [17] framed their principles with the specific intention of guiding engagement with diverse stakeholders as an integral step in the research process. Finally, the Bill and Melinda Gates Foundation [19] centred their principles around the practice of data sharing.

The methods used to develop principles varied in their degree of rigour, participation, and transparency. Five of the nine guiding documents cited some form of expert consultation as their methodological approach [7,11,17,20,21]. All but one of these five [20] described the purpose and rationale of these consultations in some detail. One author group described using a mixed-methods approach (gathering and triangulating insights, semi-structured one-on-one interviews, focus groups, and surveys) [7]. Three of the groups included clear and well-justified descriptions of why and how they used workshops, focus groups, and team discussions to develop and reach consensus on content [11,17,21]. All five sets of principles that employed some form of expert consultation explicitly stated their efforts to include Global South partners in dialogue surrounding their principles [7,11,17,20,21]. Three of these five sources clearly involved collaboration and consultation with partners directly involved in the research (such as principal investigators, collaborators, students, research assistants, stakeholders, etc.) [7,11,17]. Only one source described consulting community stakeholders and civil societies in the field where they were working [17].

Consultations and original data collection interviews were not the only strategy employed to develop principles. The principles proposed by Steenhoff, Crouse, Lukolyo, Larson, Howard, Mazhani et al. were co-authored by an expert panel of global health clinicians, researchers, and educators from six different countries [24]. Two sets of principles were developed based on the authors’ respective experiences working in the field (Zambia [22], and a conglomerate of countries from a career of research experiences [16]).

All sets of principles were directed at research involving partnerships across Global South and Global North countries and organizations, but the intended relevance across regions did not automatically imply inclusion of authors from both the Global South and the Global North. Of the nine sets of principles reviewed, only four were written with authorship from both Global South and Global North partners [7,17,21,24]. One source was written with only authors from the Global South [16] and three were written by authors from the Global North only [11,20,22]. For a summary of sources involving North-South consultation and authorship, refer to Table 3.

**Table 3.** Participation of actors in global North and global South in development of each source

Source	North-South Consultation	Source Authorship
Bill & Melinda Gates Foundation [19]	No	Unknown
Canadian Coalition for Global Health Research (CCGHR) [11]	Yes	Global North only
Costello and Zumla [22]	No	Global North only
Larkan et al. [7]	Yes	Global South-Global North
Netherlands Development Assistance Council (RAWOO) [21]	Yes	Global South-Global North
Raza [16]	No	Global South only
Rethinking Research Collaborative (RRC) [17]	Yes	Global South-Global North
Steenhoff et al. [24]	No	Global South-Global North
Swiss Commission for Research Partnerships with Developing Countries (KFPE) [20]	Yes	Global North only

### Shared ideas identified across sets of principles

There is significant variability in the language employed within each guiding document. While some sources include the same named principles, the ways in which these principles are defined often differ between sources (for a summary of principles and their definitions in each source, refer to Appendix B). The contrary is also true for many documents; different terms are used to describe otherwise identical principles. For example, one of the most frequently cited principles in these nine sets, both in term and definition, was ‘equity.’ Using a global health lens, ‘equity’ can be broadly defined as ‘the absence of systematic differences in health, between and within countries, that are avoidable by reasonable action’ [25, p.e1001115], and ‘are also considered unfair and unjust’ [26, p.219]. Three guiding documents defined equity in this way [7,11,24]. This definition of equity [25] was observed to align almost perfectly with the definition offered for the principle of ‘proportionality’ in the Bill and Melinda Gates Foundation [19] guiding document ‘Global Health Data Access Principles.’ This difference in language but similarity in definition demonstrates how different terminology can be used to refer to effectively the same principle. Additionally, because principles are presented in these documents as normative ideas – even where definitions are similar across documents – this does not preclude competing normative interpretations. Other examples of definitionally ubiquitous terms include agenda-setting, which is respectively referred to as ‘focus,’ ‘set agenda together,’ and, ‘setting baseline goals and objectives’ by Larkan, Uduma, Lawal, and van Bavel [7], KFPE [20], and Raza [16]. Furthermore, ‘development of national research capacity’ [22] and ‘enhanced capacities’ [20] were used to describe core commitments to capacity building. When grouped in this way according to general idea or core concept outlined in the definition, many principles appear in more than one of the nine sources (see Table 4).

**Table 4.** Principles of global health research that are similarly defined can be grouped together into themes

Theme	Principle(s)	Source
Mutual Benefits	Reciprocity	Bill and Melinda Gates Foundation [19]
	Benefit	Larkan et al. [7]
	Writing and publishing together	Raza [16]
	Mutual Benefits	Steenhoff et al. [24]
	Share data and networks	KFPE [20]
	Pool profits and merits	KFPE [20]
	Shared benefits	CCGHR [11]
Agenda Setting	Focus	Larkan et al. [7]
	Setting baseline goals and objectives	Raza [16]
	Setting up future milestones of the project	Raza [16]
	Set agenda together	KFPE [20]
Equity	Equity	Larkan et al. [7]
	Equity	Steenhoff et al. [24]
	Responsiveness to causes of inequities	CCGHR [11]
	Proportionality	Bill and Melinda Gates Foundation [19]
Accountability	Accountability	Bill and Melinda Gates Foundation [19]
	Informing each other and following rules and regulations	Raza [16]
	Account to beneficiaries	KFPE [20]
Capacity Building/ Strengthening	Development of national research capacity	Costello & Zumla [22]
	Enhance capacities	KFPE [20]
	Strengthening capacity for conducting socially relevant research should be a specific aim of the partnership	RAWOO [21]
Sustainability	Sustainability	Steenhoff et al. [24]
	Commitment to the future Invest in relationships	CCGHR [11] RRC [17]
Define Roles	Sharing and assigning responsibilities	Raza [16]
	Clarify responsibilities	KFPE [20]
Engage Stakeholders	Interact with stakeholders	KFPE [20]
	A broad-based consultative process, however painstaking and time-consuming it may be, should proceed any program	RAWOO [21]
Understand the Context	Values	Larkan et al. [7]
	Critically engage with context	RRC [17]
Actionable Research	Emphasis on getting research findings into policy	Costello & Zumla [22]
	Apply results	KFPE [20]
Communication	Communication	Larkan et al. [7]
	Effective communications	Raza [16]
Data Access	Disseminate results	KFPE [20]
	Rules and norms for sharing and handling data	Raza [16]
Humility	Humility	Steenhoff et al. [24]
	Humility	CCGHR [11]
Inclusivity	Inclusivity	Steenhoff et al. [24]
	Inclusion	CCGHR [11]
Mutual Learning	Promote mutual learning	KFPE [20]
	Keep learning	RRC [17]
Social Justice	Social justice	Steenhoff et al. [24]
	Put poverty first	RRC [17]
Transparency	Disclosing financial interests	Raza [16]
	Commit to transparency	RRC [17]
Trust	Authentic partnerships Mutual trust & shared decision making	CCGHR [11] Costello & Zumla [22]
Principles with distinct definitions*	Adapt and respond	RRC [17]
	Leadership	Larkan et al. [7]
	National Ownership	Costello & Zumla [22]
	Prevention of adverse impact	Steenhoff et al. [24]
	Promotion of common good	Bill and Melinda Gates Foundation [19]
	Redress evidence hierarchies	RRC [17]
	The Northern partner should be prepared to relinquish control and accept considerable autonomy on the part of the Southern partner	RAWOO [21]
	Resolution	Larkan et al. [7]
	Respect	Bill and Melinda Gates Foundation [19]
	Respect diversity of knowledge and skills	RRC [17]
	Secure outcomes	KFPE [20]
	Stewardship	Bill and Melinda Gates Foundation [19]

* These principles cannot be grouped into any particular theme; rather, they each have their own distinct meaning and definition

Interestingly, no single principle appeared across all nine sources. That said, all nine sets included principles concerned in some fashion with fairness, equity, or justice in research, although ideas about how this fairness is achieved and in what ways (i.e. by mutual sharing of benefits or shared agenda setting) differed. The most commonly referenced principles were ‘mutual benefits,’ (six instances [7,11,16,19,20,24]) and ‘equity’ (four instances [7,11,20,24]). A complete list of principles and the number of guiding documents including each of these is provided in Table 5.

**Table 5.** Number of sources citing each principle (organized by definition according to theme)

Principle (by theme)	Number of sources that include it
Mutual Benefits	6
Equity	4
Accountability	3
Agenda Setting	3
Capacity Building/Strengthening	3
Sustainability	3
Define Roles	2
Engage Stakeholders	2
Understand the Context	2
Actionable Research	2
Communication	2
Data Access	2
Humility	2
Inclusivity	2
Mutual Learning	2
Social Justice	2
Transparency	2
Trust	2
Adapt and respond	1
Leadership	1
National Ownership	1
Prevention of adverse impact	1
Promotion of common good	1
Redress evidence hierarchies	1
Relinquish Control	1
Resolution	1
Respect	1
Respect diversity of knowledge and skills	1
Secure outcomes	1
Stewardship	1

## Discussion

### Choosing global health principles to guide transnational research partnerships

Given the number of different principles and sets of principles that exist to guide global health research partnerships, the question arises: which of these sets, or combination of these, is best suited to guide a given research project? While each set of principles was uniquely developed with different priorities in mind, research teams should consider the manner in which principles were established when selecting which principles should inform their respective partnership practices.

Fundamentally, partners should ensure that the principles they apply in their research were developed by partners in both the Global South and the Global North. This is arguably necessary if the resources employed to build and evaluate partnerships are to meaningfully reflect the principles they endorse (e.g. inclusivity, equity, accountability). Jenstch and Pilley suggest that when partners in the Global North reflect on their North-South partnership practices, they tend to (often unintentionally) emphasize principles rooted in paternalism [12]. Further, ideas originating in the Global South are often given less attention, viewed with less confidence, or presented as influenced by northern perspectives [27]. Partnerships looking to work against this precedent, and to establish or sustain ‘good’ global health research partnerships, should seek to apply principles that have been developed collaboratively by diverse actors, thereby giving equally valued attention to perspectives from both the Global South and the Global North. Doing so provides a starting point from which transnational research teams can begin to discuss what is important to them in a partnership, and work to overcome power inequities that intrinsically exist.

### Utilizing global health research principles

While the inclusion of principles developed collaboratively by researchers in the Global South and the Global North can assist in the redistribution of power, it is important for partnering institutions to recognize that these principles do not guarantee the implementation of equitable and power-attentive practice. In their examination of what constitutes a ‘valuable international global health partnership,’ Yarmoshuk, Guantani, Mwangu, Cole, and Zarowsky found that power imbalances existed not only in North-South partnerships, but in North-North and South-South partnerships as well [28]. This finding suggests that the involvement of multiple contextual perspectives in the formation of partnership values is insufficient to prevent power dynamics from threatening the integrity of a partnership (e.g. the presence of one North and one South partner in the partnership). Partnering institutions must acknowledge that it is the actual implementation of chosen principles – not only their discussion or consideration – that establishes a precedent for power redistribution in a given project [29].

While the principles presented in this review provide a useful starting point for teams to begin thinking critically about their research partnerships, there is a general lack of guidance available on how these principles can and should be integrated into practice. There is also a lack of accountability to incorporate ‘good’ research principles and guidance on how principles should be ‘weighted’ in relation to one another. It is possible that the implementation of some principles may interfere with the success of others. Best practices for implementing principles simultaneously are needed to understand how they work together to form a ‘good’ global health research partnership.

Certain resources [19,20,22] provide ‘benchmarks’ or discussion questions as markers of whether principles are present within partnerships. Teams looking to change their research practice should use other resources in the implementation of global health research partnership principles. Implementation of these principles could be facilitated by a number of existing tools meant to support the development and sustainment of transnational research partnerships [29–34].

### Reaching consensus on defining principles

The importance of understanding the use of different terms to refer to the same principles cannot be understated. Different terms can be associated with different values, and these associations can have real effects on how research partnerships are designed and implemented. As previously suggested, the existence of several definitions for the same term also poses a challenge for global health researchers looking to initiate or evaluate transnational research partnerships and projects within the contexts of specific sets of principles. If as a field we cannot reach consensus on what we mean by key guiding principles, how can we expect to successfully implement them? While studies have been conducted to illustrate what these principles might look like in practice [8,21,23], no consensus on explicit definitions has been reached.

Additionally, the fact that no one principle is ubiquitously acknowledged in each of the nine sources reviewed suggests that there is both a lack of agreement on how seemingly identical principles are defined and which should be prioritized. This lack of agreement may be attributed to the interdisciplinary nature of global health work. With the broad engagement of so many disciplines, it can be expected that one field may use a term (or understand it) differently than another. While these differences may prevent absolute clarity and uniformity between disciplines, they cater to a degree of flexibility and independence that is important for partners working in resource-varied settings with unique working relationships. Given these considerations, future global health discussions should move towards critically examining (using recent case work) frequently used buzzwords and establishing disciplinary definitions. In doing so, global health leaders can attempt to identify broad categories of normative consideration that must be addressed in research partnerships. One example of this could be use of the word ‘fairness’ to refer broadly to both equity and mutual benefits in global health partnerships. Without prescribing what fairness should entail, the inclusion of such a principle should prompt those in the partnership to interrogate what fairness means and requires in their independent context.

### Equity as a dominant value in global health research

Based on the recurring use of the term ‘equity’ and the emphasis placed on this term in the documents reviewed, it is clear that equity exists less as a ‘principle’ for global health work and more as a shared vision, fundamental goal, or encompassing value. For example, ‘capacity strengthening’ [20–22] in both the Global South and the Global North contributes to the improvement of health and research systems in lesser-resourced areas, while bi-directionally narrowing the knowledge gap between the South and the North. Similarly, ensuring ‘mutually beneficial’ [7] partnerships exist is a direct action to prevent avoidable knowledge or resource disparities. For those conducting research in global health, the concept of equity is more than simply a project goal; it is also a qualitative standard to which research practice must be held [13]. The effectiveness of any strategies to establish equity may be difficult to conceptualize in the absence of evaluation. How can efforts to support equity within specific research partnerships be measured or otherwise evaluated? Further discussion on what it means to effectively support equity in the context of global health research is needed. Such discussion and debate may also help clarify existing and reveal other yet to be identified principles of ‘good’ transnational research partnerships.

## Limitations and future directions

While we are confident our search returned key sets of principles developed to support global health research practices, a scoping review is not a systematic review, and grey literature hand-searching is inherently imperfect.

It is possible lesser-known global health organizations or groups without a web presence or publishing in languages other than English have developed additional sets of principles to guide their global health research partnerships. We encourage future research teams to endeavor a systematic review of guiding principles that includes sources that are not in English, and sources that are potentially not available online. Future reviews could include partnership principles from outside of traditional global health settings as they may confer important non-disciplinary specific learnings.

## Conclusion

This review identified and provided a summary of methods used as well as content within nine sets of principles developed to support global health research partnerships. While each of these sets of principles constitutes a useful starting point from which partnering institutions may start to think about their transnational research practice, some cautions and considerations are merited. Some sets of principles were developed without clear processes of international consultation or input from actors in the Global South. These may be less helpful or appropriate for those who value working with vantage points informed by both the Global South and the Global North actors. It is important to recognize that for every principle of global health research, many disciplinary-informed definitions exist across documents. It may be helpful for research teams and institutions to hold explicit discussions to clarify what exactly is implied by a commitment to, for example, ‘mutual benefits’ or ‘equity.’ Beyond clarifying the requirements for principle implementation in a particular research partnership, there is also the question of whether and how a team or institution will know if they have been successful in upholding a commitment to particular principles. Is there a plan to track, measure, or otherwise evaluate the effective implementation of principles within the partnership? Highlighting differences, similarities, and strategies for implementation associated with specific principles of ‘good’ global health research partnerships will support the actualization of those principles, and constitute valuable work for future researchers invested in improving transnational research partnerships.

## Paper context

Many documents exist proposing ‘principles’ to guide global health research partnerships. This article consolidates these principles, comments on commonalities and differences between documents, and proposes criteria for choosing principles to guide a partnership. Researchers should choose a set developed in consultation with partners from the Global North and South. Tools are needed to support implementation of these principles. Further discussion is needed to reach consensus on what principles are integral to global health research partnerships.

## Appendix A.

**Table A1.** List of global health research funders’ websites hand-searched for grey literature

Organization Name	Website
Bill and Melinda Gates Foundation	www.gatesfoundation.org
Canadian Association for the study of International Development (CASID)	www.casid-acedi.ca
Canadian Coalition for Global Health Research (CCGHR)	www.ccghr.ca
Council on Health Research for Development (COHRED)	www.cohred.org
Centre for Disease Control and Prevention (CDC)	www.cdc.gov
Elhra	www.elrha.org/programme/research-for-health-in-humanitarian-crises/
European Research Council	erc.europa.eu
Ford Foundation	www.fordfoundation.org
Foreign, Commonwealth & Development Office	www.gov.uk/government/organisations/foreign-commonwealth-development-office
German Federal Ministry of Education and Research	www.bmbf.de/en/index.html
Global Affairs Canada	www.international.gc.ca
International Development Research Centre (IDRC)	www.idrc.ca/en
National Institutes of Health	www.nih.gov
Canadian Red Cross	www.redcross.ca
United Nations High Commissioner for Refugees (UNHCR)	www.unhcr.org
United Nations International Children’s Emergency Fund (UNICEF)	www.unicef.org
Wellcome Trust	www.wellcome.ac.uk
World Health Organization (WHO)	www.who.int

## Appendix B.

**Table B1.** Principles to guide global health research partnerships

Source	Principles	Definition
Bill & Melinda Gates Foundation [19] “Global Health Data Access Principles”	Promotion of common good	“Data access should enhance the value of research and program effectiveness.” [p.2]
	Respect	“Respect must be given to matters of identity, privacy, and confidentiality as they pertain to the individuals and communities from or about whom data are collected. Respect must also be given to matters of attribution as they pertain to researchers, evaluators, and their collaborators.” [p.2]
	Accountability	“All processes and procedures for data access will be transparent, clear, and consistent with data management standards that ensure quality data, appropriate security, and equitable access.” [p.2]
	Stewardship	“All who produce, share, and use data are stewards of those data. They share responsibility for ensuring that data are collected, accessed, and used in appropriate ways, consistent with applicable laws, regulations, and international standards of ethical research conduct.” [p.2]
	Proportionality	“The needs of investigators must be balanced against those of communities and sponsors that expect benefits to arise from the activities to which they contribute information or resources.” [p.2]
	Reciprocity	“The aim of benefiting the individuals and communities who enable and support inquiry should be furthered to the extent possible and is of particular importance when involving individuals and communities from developing countries.” [p.2]
Larkan et al. [7] “Developing a Framework for Successful Research Partnerships in Global Health”	Focus	“Common goals and minimum common programme … shared interest and vision.” [p.4]
	Values	“ … understanding the organisational culture of each partner and the underlying societal norms within which each partner operates.” [p.4]
	Equity	“ … recognition of, and respect for, differing capacities; and a sharing of resources such that inclusion occurs on an equitable basis.” [p.4]
	Benefit	“ … reciprocal and mutually beneficial relationships among all partners. Such benefits may include the generation of skills, rewarding experiences, knowledge exchange etc.” [p.4]
	Communication	“ … transparent; open; honest; consistent; unambiguous, and effective” [p.4]
	Leadership	“ … delegation of roles and responsibilities, but also management and accountability. In particular, balance and diplomacy, when dealing with all collaborators in the partnership, were identified as essential.” [p.4]
	Resolution	“Firstly, there should be an acknowledgement that partnerships may encounter difficulties, and resolve, perseverance, and determination will be required to deal with any such difficulties. Secondly, while the on-going processes of mediation and conflict resolution may offer solutions, the need for the dissolution of partnerships may still ensue.” [p.4]
Raza [16] “Collaborative Healthcare Research: Some Ethical Considerations”	Effective communication	“Communication should be established at all levels, sharing expertise, data, chemicals and other necessities.” [p.181]
	Setting baseline goals and objectives	“Each partner must lay down his or her contribution to the main goals of the collaborative project.” [p.181]
	Sharing and assigning responsibilities	“Determining clear role of each collaborating team member will reduce stress, enhance performance and clarify mutual expectations.” [p.181]
	Setting up future milestones of the project	“After an initial framework of different activities is set and agreed upon by the team leaders, the next step is to estimate a time limit to each of them and make a tentative timetable for different activities until the termination of the project.” [p.181]
	Rules and norms for sharing and handling data	“The team heads must mutually discuss and decide, set practical rules and communicate to their subordinates the policies related to data ownership, keeping, sharing, disclosure and publication while the project is in progress and after it has ended.” [p.182]
	Writing and publishing together	“ … a mutual understanding about the mechanism as to how they will write and publish together once different team members start doing experiments, generate valid data or perform data-analysis.” [p.182]
	Disclosing financial interests	“ … decide that if any expected or unexpected results lead to financial gain, patent or intellectual property, how the interest will be decided among themselves and those who funded the project.” [p.182]
	Informing each other and following rules and regulations	“The team members in collaboration must respect and follow rules and regulations as set by the funding agencies or grantee institutions or the nature of their research. They should inform each other about their own limitations or claims with regards to handling of materials, use or transfer of equipment, confidentiality of clinical data, budgeting, intellectual property rights etc.” [p.183]
Steenhoff et al. [24] “Partnerships for Global Child Health”	Equity	“ … absence of systematic disparities in controllable or remediable aspects of health between groups with different levels of underlying social advantage with respect to wealth, power, or prestige.” [p.4]
	Inclusivity	“include[s] promoting the involvement and participation of all major stakeholders, particularly communities who may be disadvantaged by poverty, low education, race, or other factors.” [p.6]
	Sustainability	“ … refers to building a long-term vision for strengthening child health while working to conduct successful short-term activities.” [p.6]
	Mutual Benefits	“A reciprocal and mutually beneficial relationship … which may involve outlining reconciling objectives that are not strongly shared while ensuring that objectives are not divergent.” [p.6]
	Prevention of adverse impact	“ … taking steps to minimize adverse outcomes to visiting providers, students, and trainees as well as to patients, communities, local providers, and health facilities in LMICs.” [p.7]
	Social justice	“The principle of social justice calls for partners to work together to value diversity (including gender, religion, age, race, social class, socioeconomic circumstance or disability, and sexual orientation); recognize social, historical, political, economic, and environmental determinants of health; and seek ways to mitigate inequities.” [p.7]
	Humility	“ … calls on stakeholders to dedicate efforts to understand their own assumptions, biases, and differing values and to center the partnership on the act of learning rather than on knowing.” [p.7]
Costello & Zumla [22] “Moving to Research Partnerships in Developing Countries”	Mutual trust and shared decision-making	“Do the partners know each other well and trust each other? Do the partners have regular and easy communications? Do the partners have good access to databases and information from international organisations? Who proposed the research programme? Do all participants understand it? Did people who will be affected by the research participate in developing the research theme? Were users consulted? Are the likely beneficiaries of the research clearly defined?” [p.829]
	National Ownership	“Do national partners have overall administrative responsibility and responsibility for scientific supervision? If not, why not? Is there transparency, with equal access of partners to scientific and budgetary documents and fund allocation decisions? Do the national partners have adequate training and audit systems to take full responsibility for programme implementation? Are there clear and fair rules about who has authority over financial decisions? Will the partners share equally in any findings or potential commercial value, and has an agreement been made?” [p.829]
	Emphasis on getting research findings into policy	“Does the research give due consideration to the social, political, economic, and technical situation of the partners? Is traditional knowledge and custom incorporated into the research plan? Is there a dissemination plan? Does this include publications or reports for the people directly affected by the research and by a wider audience than the scientific community? What is the plan about targeting government and non-governmental policymakers, stakeholders, and opinion leaders? Is authorship of scientific publications balanced? What steps are being taken to ensure that research findings will quickly be put into practice?” [p.829]
	Development of national research capacity	“Does the research fit into existing national or regional research policy? Is the collaboration being monitored and evaluated both internally and externally? Are national partners properly represented in evaluations? How will the partnership develop local research capacity in the field of interest? Who will receive training, where, and for how long? How will South-to-South collaboration be promoted? What will happen to staff when existing research projects finish? Will this research partnership reduce the migration of researchers to the developed world or into the bureaucracies of international agencies? How will the partner institution sustain research and continue research after the programme is finished?” [p.829]
Swiss Commission for Research Partnerships with Developing Countries (KFPE) [20] “A Guide for Transboundary Research Partnerships”	Set agenda together	“Determining research questions, research approaches, and research methods jointly is a first important step towards more equity in cooperation, shared ownership and mutual trust.” [p.3]
	Interact with stakeholders	“Ideally, researchers should involve important stakeholders early on, in the formulation of research questions or even in certain research activities. The more specific the research is in terms of addressing political and societal issues and users’ needs, the more relevant – and likely to be used – the research results will be.” [p.4]
	Clarify responsibilities	“Any partnership ultimately depends on each partner contributing what they are particularly skilled in doing. Dividing the work makes it necessary to clarify and assign the responsibilities of the partners involved and, based on this, their rights and obligations.” [p.5]
	Account to beneficiaries	“Answering to particular expectations of potential beneficiaries of research is thus an obligation, but also an effective means of communication and feedback. Being accountable «downward» to a specific group of beneficiaries can trigger an important echo, leading to enhanced and genuine partnerships, new research questions and, last but not least, to broader and deeper dissemination of results” [p.6]
	Promote mutual learning	“The willingness of those involved to engage in dialogue and learning processes is a crucial precondition for generating added value at the institutional level.” [p.7]
	Enhance capacities	“ … the focus is on increasing both knowledge and know-how, while at the same time fostering the capacities of all parties involved, including all stakeholders and junior scientists. Both processes should enhance each other.” [p.8]
	Share data and networks	“ … in North-South partnerships, as a rule, knowledge and information are not distributed one-sidedly: both sides have information and relationships that are crucial for the success of their joint research project. Negotiating the «give and take» can lead to a win-win situation.” [p.9]
	Disseminate results	“Every researcher must therefore disseminate his or her findings in forms that enable potential users to find, understand, and use them.” [p.10]
	Pool profits and merits	“This includes equal acknowledgement of authors as well as selection of a publication channel that caters to all interests. Profit distribution can be free of conflicts in cases where investors have achieved their goals and researchers have been able to publish their work as desired and agreed upon.” [p.11]
	Apply results	“ … effective implementation of research results means speaking the language of the users and presenting the results in such a way that they have a meaning for users.” [p.12]
	Secure outcomes	“Integration into research networks (including South-South cooperation), targeted capacity development, and enhancing visibility by means of publications are some of many possible entry points for loosening dependencies and creating continuity.” [p.13]
Netherlands Development Assistance Research Council (RAWOO) [21] “North-South Research Partnerships: Issues and Challenges”	Strengthening capacity for conducting socially relevant research should be a specific aim of the partnership	“Strengthening capacity means addressing the management of the research institution, the relationship between researcher and society, and the relationship between the research institution and society. The work plan should describe the concrete activities undertaken for this purpose, such as financial support, training, policy dialogue and advocacy, as well as activities undertaken by Southern partners to ensure recognition in their own society. Training a few PhD students or purchasing some equipment is not sufficient.” [p.29]
	The Northern partner should be prepared to relinquish control and accept considerable autonomy on the part of the Southern partner	“Ideally, the Southern partner plays an autonomous role in shaping the partnership. It chooses its research partners from the North and decides which elements of the programme will require cooperation with Northern researchers. It also decides which type of expertise it wants from the Northern partner, on which scale, and at which level: junior or senior. At the same time, Southern partners have to take into account the demands which the Northern partners are required by their own institutions to meet.” [p.29]
	A broad-based consultative process, however painstaking and time-consuming it may be, should proceed any program	“This process should ensure that the motives for establishing the partnership are clear to all stakeholders and that the objectives of the partnership are well defined and clearly communicated between the collaborating institutions. This paves the way to the development of mutual trust.” [p.30]
Canadian Coalition for Global Health Research [11] “CCGHR Principles for Global Health Research”	Authentic partnering	“Authentic partnering is about ensuring our intentions and actions as global health researchers are aligned around equitable research relationships, processes, and outcomes. It involves creating and maintaining a strong foundation of trust.” [p.4]
	Inclusion	“Commitment to inclusion invites those involved in [global health research] to promote equity by proactively and intentionally providing opportunities for diverse people to be engaged in research processes. The principle of inclusion also challenges those involved in [global health research] to seek diverse perspectives in the definition of research questions, formation of research teams, or creation of research initiatives.” [p.5]
	Shared benefits	“The principle of shared benefits is about collectively striving to share emerging benefits, knowledge, evidence, and innovations in equitable, openly accessible ways. It invites us to find ways to prioritize equity amongst all those involved in research in the distribution of benefits. This extends to all players in research, including trainees and research participants.” [p.6]
	Commitment to the future	“Our commitment to the future is about honouring our global citizenship and investing in a better, more equitable world where human rights, including the right to health, are protected and promoted.” [p.7]
	Responsiveness to causes of inequities	“People involved in [global health research] should be aware of the historical, social, cultural, political, economic, and environmental reasons for health inequities. They should strive to understand health inequities as inseparable from issues of power.” [p.8]
	Humility	“The principle of humility is about positioning ourselves in a place of learning rather than knowing.” [p.9]
Rethinking Research Collaborative (RRC) [17]	Put poverty first	“ … partners need to constantly question how the process and activities of the research are addressing the end goal. This requires a consideration of whose knowledge and agendas count and greater attention to research uptake and use long after initial funding might end.” [p.9]
	Critically engage with context	“A commitment to this principle requires conscientious analysis of the contexts of research governance, implementation and use. This should include a systematic mapping of the relevant stakeholders, as well as consideration of the representativeness of both partnerships and agenda-setting/evaluation committees and review colleges.” [p.9]
	Redress evidence hierarchies	“ … funders, brokers and partners should recognise that different stakeholders (including those from different academic traditions as well as other development professionals) will have different expectations as to what “quality evidence” means to them. This influences whose knowledge is valued, how research is designed and implemented, what types of research outputs are produced and which audiences are considered.” [p.10]
	Adapt and respond	“ … every actor should take an adaptive approach that is responsive to context; constantly review and renegotiate all the research parameters.” [p.10]
	Respect diversity of knowledge and skills	“To live up to this principle requires time to be taken at the outset to explore the knowledges, skills and experiences that each partner brings and contributes to making the partnership greater than the sum of its parts. All contributions should be made explicit and be respected.” [p.11]
	Commit to transparency	“ … a code of conduct or memorandum of understanding that commits each partner to transparency in all aspects of the project administration and budgeting; and that sets out clearly the rights of all partners regarding acknowledgement, authorship, intellectual property and data use.” [p.11]
	Invest in relationships	“ … significant investment in creating spaces for new partnerships to emerge and for existing relationships to develop and sustain through funded time for meaningful communication.” [p.11]
	Keep learning	“ … constant critical reflection and learning within and beyond the partnership.” [p.12]
